# Editorial: Community engagement: models and effectiveness

**DOI:** 10.3389/fpubh.2026.1818521

**Published:** 2026-03-25

**Authors:** Susan M. Swider, Lori Edwards, Mallory Bejster

**Affiliations:** 1 Department of Community, Systems and Mental Health, Nursing, College of Nursing, Rush University, Chicago, IL, United States; 2 Department of Family and Community Health, School of Nursing, University of Maryland, Baltimore, MD, United States

**Keywords:** CHWs, community based participatory research, community engagement (CE), community engagement measures, community engagement methods, community participation, health equity

The Centers for Disease Control (CDC) defined the term Community engagement (CE) as “the process of working collaboratively with groups of people who are affiliated by geographic proximity, special interests or similar situations with respect to issues affecting their wellbeing” (1). This Research Topic was designed to highlight current work in the area of CE in health promotion and disease prevention interventions. We were particularly interested in measures and methods of community engagement, in both public health practice and research. We received 37 complete submissions of which we accepted 28. Of those accepted, 43% were Original Research, 32% were Community Case Studies, and the remainder were Perspectives (11%), Brief Research Reports (7%), Methods (3.5%), and Clinical Trials (3.5%). The number and quality of submissions were reflective of the growing importance of CE work in both research and public health/health promotion practice. The editors would have liked more Community Case Studies, with robust evaluation, to serve as evidence for others in practice in best methods for engaging populations of focus.

## Populations

As expected, the majority of the articles were focused on populations experiencing disproportionate health risk due to structural, historical, and systemic inequities and barriers. Community engagement is often discussed as an important method to partner with populations who have experienced such inequities and barriers to co-creating more equitable, inclusive and effective health promotion efforts. For this Research Topic, 39% of the articles focused on work being done with non-Western or developing nations, including Sri Lanka, Ethiopia, Benin, India, Ghana, South Africa, and China. Of the remaining articles, 58% were set in the U.S. and focused on underserved and marginalized populations across a variety of regions including rural youth; Latino/a, Native American, and Black/African American populations; the LGBTQ+ community; immigrant and refugee populations; those living with HIV; those with substance use disorder; and those at risk of diabetes, cancer, obesity, and inadequate access to care. The remaining article looked at TB control across many countries and populations. The populations of focus in this Research Topic are largely populations who experience structural and systemic barriers to maximizing health, so the need for meaningful and equitable methods of engagement is critical. It is important that future CE work continue to focus on a wide range of populations, because there is a need across the spectrum of populations and countries for interventions and research that are meaningful and accessible to each community/population.

## Focus of work

The focus of much of the work was on describing and/or testing methods of engaging with communities. In many cases, the engagement was focused on increasing community knowledge of or participation in ongoing research, in order to increase the validity, reliability and usefulness of the results. Many articles described methods of engaging the community, sometimes with descriptive results of their effectiveness included. This is often the strategy used in community based participatory research, but it was valuable to see the specific ways engagement was used. A few articles took a broad view of the issue of CE and looked at results across disease entities, such as tuberculosis (Kumah), Buruli ulcer (Narh et al.) or populations such as rural youth (Grenon et al.). D'Alonzo et al. described a new method of CE and Lemma et al. used a CE method with Ethiopians learning to manage livestock more effectively. Nicholas et al. looked at whether current CE initiatives were building community capacity.

A number of studies used CE as part of their research methods to engage participants or help with development of valid and reliable interventions and measures. Several articles described methods for working with populations of focus on intervention development (Khatib et al., Humphries et al., Sharma et al., Kang et al.). Other authors looked at participant satisfaction with participating in community initiatives, either measured via participation (Syed et al.) or self-reported satisfaction (Wolfe et al., Naaseh et al., Yang et al.). Banks et al. looked at methods of increasing participation and retention in research amongst Black/African American female teens. And several studies looked at the results of CE methods on their study outcomes, such as improved child development scores (Syed et al.) and increased CHW knowledge of handwashing (Wouékpé et al.). Li and Yu described how community group buying initiatives developed to become community mutual aid efforts during the Covid pandemic. Islam et al. measured the impact of youth led interventions in Sri Lanka on adult self-rated health and happiness.

A number of articles were Community Case Studies of their CE efforts, including such work as recommending a new CE method of support service delivery (Carman et al.), public awareness campaigns (Narh et al.), or using community asset mapping with a One Health approach to help communities strengthen their workforce in a comprehensive manner (Jules et al.). McGladrey et al. used precision public health methods to map colorectal cancer screening needs in their community and Cadzow et al. and Narh et al. also used CE methods to increase screening for diabetes and sexually transmitted infections respectively. Wanje et al. used CE methods to help pregnant women research participants understand and participate with sample collection and Morrison et al. used CE methods with Montagnard youth to get them involved in intervention development for their community in North Carolina. Terral et al. used CE methods to enhance a focus on health equity in their work with youth who have substance use disorders. Most notably, although there were many creative methods described here, there were no comparisons of effectiveness of methods across similar populations, and the method descriptions were conceptual, rather than detailed.

## Outcomes

The work reported here had very positive outcomes. In almost every article, descriptive statistics on participation or community satisfaction were positive. The research reported also demonstrated effectiveness of the intervention, including CE methods. It was exciting to see so many public health practitioners and researchers working to incorporate CE methods into their work, and the wide variety of CE methods being used, addressing the unique needs and concerns of the populations of focus.

The majority of the articles included here used descriptive statistics for CE measures. No articles submitted compared different CE methods or looked at measuring CE across a continuum from participation to consultation to collaboration. Additionally, it would be informative for those using CE methods to read research measuring whether CE made a difference in intervention outcomes. That is, were intervention outcomes for populations with greater degrees of engagement in developing and implementing the interventions better than outcomes for interventions without specific focus on enhancing CE? Another addition needed to the literature would be for authors to describe which aspects or methods of CE were most effective and how CE could be improved in future studies and/or interventions.

## Recommendations for future work

The work described in this Research Topic reflects a snapshot of scholarship in CE at this point in time but cannot possibly be representative of all the CE work taking place across the globe. CE as a concept has been discussed in the literature for many years but only more recently have practitioners and researchers begun to measure its effectiveness. Overall, the focus was on different methods of CE, with some descriptive evaluation of the results, that is, how many people were involved in an intervention and did they value their engagement. There were no papers included that compared the effectiveness of different methods of CE or looked at CE measures as part of a continuum, from engaging participants to having communities actively involved in identifying concerns and developing interventions to address them. An additional area of recommendation for further research is to test whether CE methods improved the effectiveness of the interventions in which they are used. These types of studies are important steps in validating the use of CE methods and enhancing uptake among public health practitioners and scholars, with the intent of improving the health of populations and advancing health equity. We look forward to moving forward from these articles to see the continued development and testing of this important concept.
